# Surfactant-Free Chitosan/Cellulose Acetate Phthalate Nanoparticles: An Attempt to Solve the Needs of Captopril Administration in Paediatrics

**DOI:** 10.3390/ph15060662

**Published:** 2022-05-25

**Authors:** Noelia Nieto González, Guido Cerri, Jesús Molpeceres, Massimo Cossu, Giovanna Rassu, Paolo Giunchedi, Elisabetta Gavini

**Affiliations:** 1 PhD Program in Chemical Science and Technology, Department of Chemistry and Pharmacy, University of Sassari, 07100 Sassari, Italy; nnietogonzale@uniss.it; 2 Department of Architecture, Design and Urban Planning-GeoMaterials Laboratory, University of Sassari, 07100 Sassari, Italy; gcerri@uniss.it; 3 Department of Biomedical Sciences, Faculty of Pharmacy, University of Alcalá, 28805 Alcalá de Henares, Spain; jesus.molpeceres@uah.es; 4 Department of Chemistry and Pharmacy, University of Sassari, 07100 Sassari, Italy; mcossu@uniss.it (M.C.); pgiunc@uniss.it (P.G.); eligav@uniss.it (E.G.); 5 Department of Medical, Surgical and Experimental Sciences, University of Sassari, Via Muroni 23a, 07100 Sassari, Italy

**Keywords:** captopril, chitosan, nanoparticles, cellulose acetate phthalate, paediatric formulation, paediatric disease

## Abstract

The Paediatric Committee of the European Medicines Agency encourages research into medicinal products for children, in particular, the development of an age-appropriate formulation of captopril is required in the cardiovascular therapeutic area. The aim of this study was the development of a liquid formulation using nanoparticles based only on chitosan and cellulose acetate phthalate containing captopril for the treatment of hypertension, heart failure and diabetic nephropathy in paediatric patients. Nanoparticles were prepared by a nanoprecipitation method/dropping technique without using surfactants, whose use can be associated with toxicity. A range of different cellulose to chitosan weight ratios were tested. A good encapsulation efficiency (61.0 ± 6.5%) was obtained when a high chitosan concentration was used (1:3 ratio); these nanoparticles (named NP-C) were spherical with a mean diameter of 427.1 ± 32.7 nm, 0.17 ± 0.09 PDI and +53.30 ± 0.95 mV zeta potential. NP-C dispersion remained stable for 28 days in terms of size and drug content and no captopril degradation was observed. NP-C dispersion released 70% of captopril after 2 h in pH 7.4 phosphate buffer and NP-C dispersion did not have a cytotoxicity effect on neonatal human fibroblasts except at the highest dose tested after 48 h. As a result, chitosan/cellulose nanoparticles could be considered a suitable platform for captopril delivery in paediatrics for preparing solid/liquid dosage forms.

## 1. Introduction

One of the roles of the Paediatric Committee (PDCO) of the European Medicines Agency is establishing and regularly updating an inventory of paediatric medicine needs in various therapeutic areas. For the cardiovascular therapeutic area, the development of an age-appropriate formulation of captopril (CAT) is required for the treatment of hypertension and heart failure as well as for the treatment and prevention of diabetic nephropathy [1]. The childhood hypertension is becoming a worldwide public health problem due to the significant increase in the paediatric population. The prevalence of arterial hypertension in children and adolescents, in apparent good health, is estimated to be about 3.5% and increases to 25% if we consider overweight and obese children [2].

CAT is an angiotensin-converting enzyme (ACE) inhibitor with a short half-life; thus, it is administered frequently during the day [3]. It is a water-soluble drug, stable as solid, but unstable in solution where CAT undergoes an oxidative degradation of the sulfhydryl group [4]. At the moment, CAT is administered using an extemporaneous liquid formulation due to its limited water stability and the absence of a suitable approved formulation for the paediatric population [5]. These extemporaneous formulations usually contain antioxidants to reduce captopril degradation. The use of extemporaneous preparation may result in a degree of content variability, which in turn may lead to a risk of toxicity due to overdosing or sub-therapeutic effects due to underdosing [6,7]. Variable bioavailability and lack of documented information about the physical, chemical and microbial stability of the preparation are also potential issues (Committee for Human Medicinal Products of European Medicines Agency, 2006). Furthermore, the off-label prescriptions of adult dosage forms are widespread to treat congenital and acquired cardiovascular disease in paediatrics [8], with a high risk of adverse events, including hospitalisation and death [9,10].

Liquid dosage forms are the favourite dosage form of the age groups 0–6 and 7–12 years [11]. The acceptability of the dosage form is crucial for achieving good patient compliance. In particular, the relevant attributes of medicine are the tastiness of the medicine, the swallowability and the practicality. Swallowing a whole solid dosage form may be problematic for children, depending on their age [12,13]. Therefore, there is a need to develop a more appropriate paediatric formulation of CAT.

In the development of formulations, knowledge of the excipient safety profile is one of the regulatory agencies’ mandatory requirements for avoiding potentially toxic or unsuitable excipients for children [14]. All excipients used in pharmaceutical formulations, and particularly those intended for use in paediatric products, should be demonstrated to be necessary for the formulation and should be used at the minimum required level. The age of the child needs to be considered when choosing the excipients to use and their use levels. In the context of this paper, whilst polysorbate 80 is widely used as a surfactant in paediatric formulations, its use can be associated with toxicity, especially in neonates [15], thus, so we investigated whether a suitable nanoparticulate formulation could be produced without the need for surfactants.

Polymeric nanoparticles (NPs) have been used or suggested to tackle paediatric formulation needs, such as developing age-appropriate formulations for each paediatric age group, mainly due to their ability to modify the physicochemical and pharmacokinetic properties of drugs loaded into them, to be easily swallowable and to facilitate easy dose adjustments as children grow [16]. Polymeric nanoparticles are therefore proposed as innovative carriers for CAT. The present work aimed to prepare nanoparticles by only exploiting the interaction between cellulose acetate phthalate (CAP) and chitosan (CH) without using surfactants to obtain a nanoparticle dispersion in an aqueous medium; this can be utilised as such or for preparing other solid or liquid dosage forms for CAT delivery in paediatrics, allowing an easy dose adjustment as children grow. CAP is a coating material characterised by pH-dependent solubility that is resistant to acidic gastric fluids, but dissolves in a mildly acidic or neutral environment [17]. In addition, CAP presents negative charge groups in its structure [18]. CH is a natural, non-toxic polysaccharide able to form an electrostatic complex with synthetic or natural polymers. CAP and CH are biodegradable and FDA-approved biocompatible polymers [19,20]. Previous authors investigated the ability of CAP and CH to form an interpolymer complex for developing packaging film [21], coating film [22], microspheres [23] and composite blends [24]. We hypothesised that CAP and CH interact due to negative charges of CAP and cationic amino groups of CH, leading to a polymeric matrix that entraps and protects CAT from chemical degradation in water. After administration, the interpolymer complex gradually dissociates in contact with biological fluids due to its pH-dependent solubility and releases CAT. As a consequence, the in vivo biodistribution/accumulation of nanoparticles and surfactants is avoided, as well as their potential toxicity in children. Thus, a formulation study was conducted to identify the correct CAP: CH polymer ratio and select the best formulation. Drug-free and loaded NPs were characterised in terms of size, PDI (polydispersity index), encapsulation efficiency, physical and chemical stability (1–28 days), zeta potential and morphology. FT-IR, X-ray diffraction and thermogravimetric analysis were also performed and cytotoxicity of the nanoparticles was evaluated on neonatal human fibroblasts (HFF-1).

## 2. Results and Discussion

### 2.1. Preparation and Characterisation of Nanoparticles

Compared to other techniques used to produce polymeric NPs, such as the commonly used emulsification and solvent evaporation method, nanoprecipitation is an easy and reproducible method that avoids the loss of drugs during preparation; surfactants are generally used for stabilising the colloidal dispersion. Nanoprecipitation is mainly employed for the encapsulation of hydrophobic drugs [25,26]. However, the preliminary results of our research have shown that it is possible to prepare NPs based on CAP and CH loaded with a hydrophilic drug (captopril) without the need for surfactants using the nanoprecipitation method–dropping technique. The absence of surfactants could contribute to a safer formulation for paediatric patients [15]. No degradation or loss of CAT was observed during NP formation since the total drug in dispersion was 100% of the drug used for NP preparation (0.5 mg/mL) (Table 1).

The mean size and PDI of NPs are shown in Table 1. The particle size of CAP:CH nanoparticles was influenced by the CH concentration. Indeed, NP-Cb showed a higher mean size than NP-Bb and NP-Ab (*p* < 0.0001); the same was true for NP-C compared to the other loaded NPs (*p* < 0.0001). These results agree with previous findings that show that the CH concentration is the most important factor affecting the NP size [27].

Whereas no significant size differences were found between the drug-free NP-Ab and NP-Bb and their drug-loaded counterparts (NP-A and NP-B (*p* > 0.05)), the loading of CAT significantly decreased the mean diameter of NP-C compared with NP-Cb (*p* < 0.0001). As reported by other authors [28,29], this phenomenon could be due to an increase in the internal cross-linking during the preparation process between CH chains in the presence of the negatively charged CAT [30], inducing a contraction of NPs. All PDI values were around 0.2, indicating a homogeneous size distribution [28].

NP-Cb and NP-C exhibited a zeta potential of +55 ± 1.08 mV and +53.30 ± 0.95 mV, respectively, due to the presence of chitosan on the surface. The high zeta potential ensures the good stability of NPs in dispersion [31].

As reported in Table 1, NP-A had a low encapsulation efficiency. The presence of CH allowed an increase in the encapsulation of CAT, with the highest encapsulation efficiency being obtained when the 1:3 *w*/*w* CAP:CH ratio was used (*p* < 0.0001). This could be attributed to the electrostatic interactions between the CAT and CH [27].

On the basis of the results obtained, NP-C dispersion was selected as the lead formulation and further characterised.

### 2.2. Morphological Analysis

**Figure 1:**
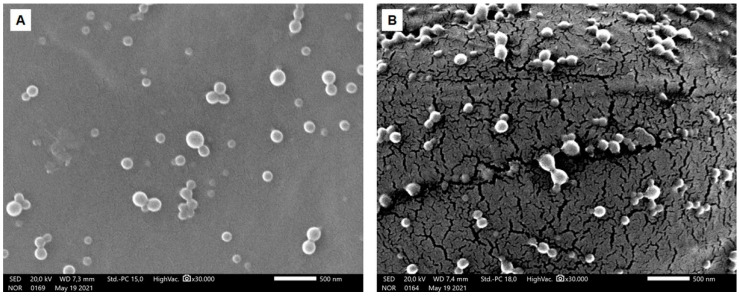
SEM images of NP-Cb (**A**) and NP-C (**B**) were acquired at 20 kV. Magnification: 30,000 times. Scale bar: 500 nm.

The morphology of NP-C and NP-Cb dispersion was observed by SEM; the pictures obtained are reported in Figure 1. NPs were spherical in shape with a smooth surface. The drug did not affect the shape of these NPs and no drug crystals were observed. NPs appear smaller than the mean diameters measured by dynamic light scattering due to the dry condition during SEM studies [32].

### 2.3. Physical Stability

The stability of the colloidal dispersion was studied by monitoring the NP size over time. Figure S1 shows the mean diameter and PDI of NP-Cb and NP-C dispersion measured over 28 days when stored at 4 °C or 25 °C. The size was stable over 28 days regardless of the storage temperature (*p* > 0.05). Additionally, the PDI did show no significant variations from day 0 to day 28, whether stored at 25 °C or 4 °C (from 0.17 ± 0.09 to 0.15 ± 0.10) (*p* > 0.05). These results confirm the good stability of NPs in dispersion, even without the presence of a surfactant due to the high zeta potential.

### 2.4. Chemical Drug Stability

**Figure 2:**
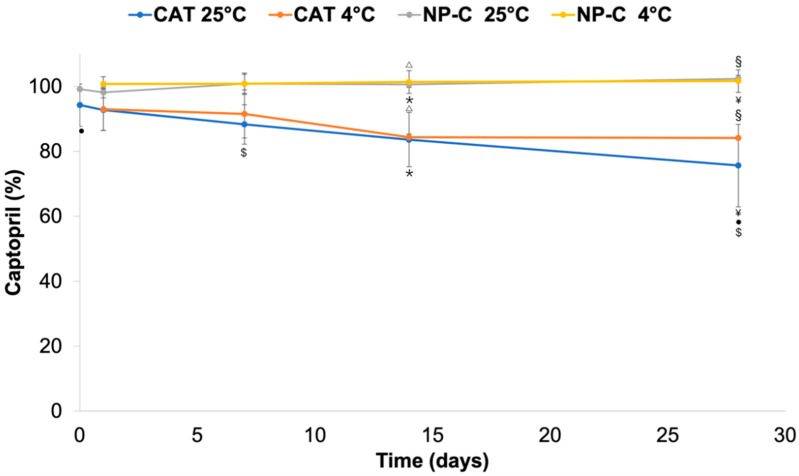
Chemical stability of CAT in aqueous solution (pH 3) and NP-C over 28 days at 4° and 25 °C. * *p* value < 0.01: NP-C day 14 at 25 °C vs. CAT day 14 at 25 °C. ¥ *p* value < 0.0001: NP-C day 28 at 25 °C vs. CAT day 28 at 25 °C. Δ *p* value < 0.05: NP-C day 14 at 4 °C vs. CAT day 14 at 4 °C. § *p* value < 0.01: NP-C day 28 at 4 °C vs. CAT day 28 at 4 °C. • *p* value < 0.01: CAT day 0 vs. CAT day 28 at 25 °C. $ *p* value < 0.05: CAT day 1 vs. CAT day 28 at 25 °C.

Simultaneously with physical stability, the chemical stability of CAT was evaluated in NP-C dispersion as compared to CAT in the aqueous solution (Figure 2). The total amount of CAT in the NP-C dispersion remained stable up to 28 days of storage both when stored at 25 °C and 4 °C (*p* > 0.05). Meanwhile, in the aqueous solution, the percentage of CAT on day 28 significantly decreased compared with CAT on day 0 (*p* < 0.01) and CAT on day 1 at 25 °C (*p* < 0.05). The main reason for CAT instability in the water solution is the oxidative degradation of the sulfhydryl group [33]. Perhaps surprisingly, degradation of CAT was avoided in the NP-C dispersion for both the encapsulated and free drug present. In other words, NP-C avoided CAT degradation of both CAT loaded in the NPs and CAT dissolved in the medium. A weak ionic interaction between the drug, negatively charged, and the positive surface charge of nanoparticles could be involved. Free-CAT in the NP-C dispersion could also interact electrostatically with free CH chains.

### 2.5. Solid State Characterisation

#### 2.5.1. Fourier Transform Infrared Spectroscopy (FTIR) Analysis

**Figure 3:**
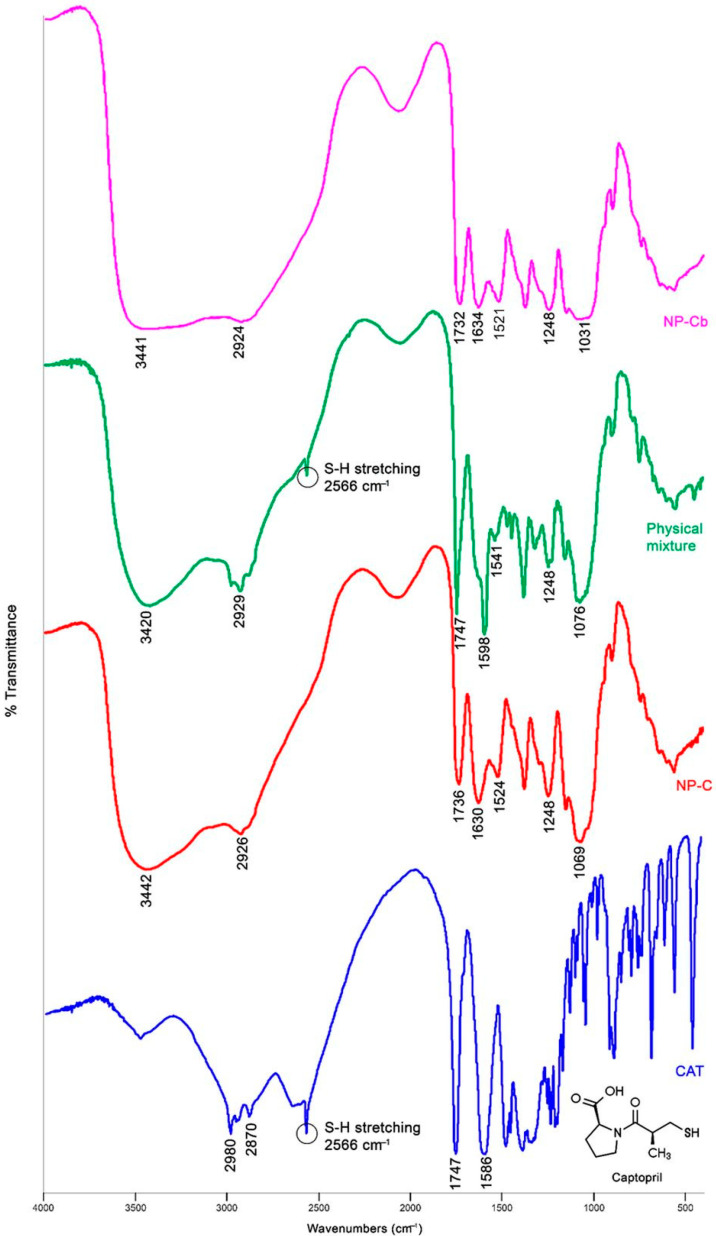
FTIR spectra of CAT, NP-C, NP-Cb and the physical mixture.

**Figure 4:**
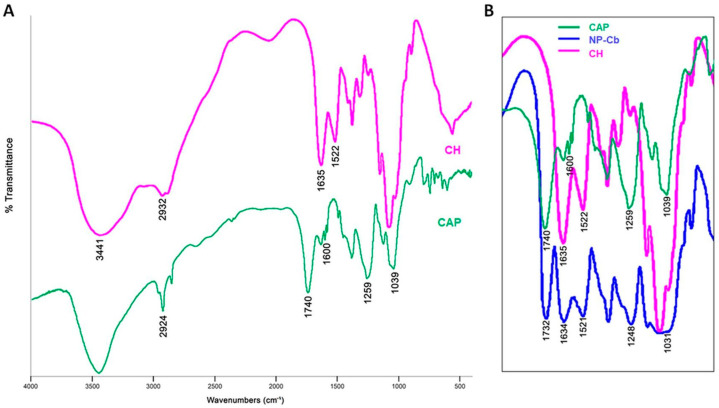
(**A**) FTIR spectra of CAP and CH; (**B**) zoom of the overlap of CAP, CH and NP-Cb spectra in the 1800–1000 cm^−1^ region.

FT-IR spectra of CAT, NP-C, NP-Cb and the corresponding physical mixture are reported in Figure 3. The spectra of CAP and CH are shown in Figure 4. In agreement with the literature, CAT shows characteristic bands at 2980–2870 cm^−1^ and 2566 cm^−1^ due to the CH_2_/CH_3_ and SH groups, respectively. The peak at 1747 cm^−1^ is related to the carboxylic group, and that at 1586 cm^−1^ to the amide [34]. The characteristic bands for CAP at 1039, 1259, 1600, 1740 and 2924 cm^−1^ are, respectively, –C–O– and –C–O–C stretching, the –C=C– conjugated vinyl aromatic ring, –C=O stretching of phthalate group of the cellulose and asymmetric and symmetric stretching of methyl groups [35]. In CH, a broad band at 3441 cm^−1^ is recognised as an OH stretching and NH vibrations. Meanwhile, a shallow band at 2932 cm^−1^ is correlated with the –CH_2_ stretching. Characteristics bands at 1635 cm^−1^ and 1522 cm^−1^ correspond to C=O stretching of the amide I band and for N–H bending of the amide II band, respectively [21,36]. NP-Cb showed a wide band between 3000–3500 cm^−1^ due to the NH and OH stretching of CH and CAP, a shift of C=O stretching (from 1740 to 1732 cm^−1^), –C–O–C– stretching (from 1259 to 1248 cm^−1^) and –C–O– stretching (from 1039 to 1031 cm^−1^) peaks of CAP, as well as an intensity increase of amide bands of CH (1634 and 1521 cm^−1^) (Figure 4B). These changes to CH and CAP spectra are indicative of intermolecular interaction between carbonyl groups of CAP and OH groups in CH and between COO– group of CAP and the amine group of CH in the polymer matrix [21,36]. The NP-C spectrum exhibits similar bands to NP-Cb, and it does not show the characteristic peak of CAT at 2566 cm^−1^ which is, however, visible in the physical mixture. This absence could be ascribed to the loading of CAT in the polymer matrix [37] or molecular interactions with the NP components that are not developed in the physical mixture and could be responsible for the shoulder at 2600–2450 cm^−1^ (Figure 3).

In the physical mixture, the peak at 1747 cm^−1^ related to the carboxylic group of CAT is also evident.

#### 2.5.2. X-ray Diffraction (XRD) Analysis

**Figure 5:**
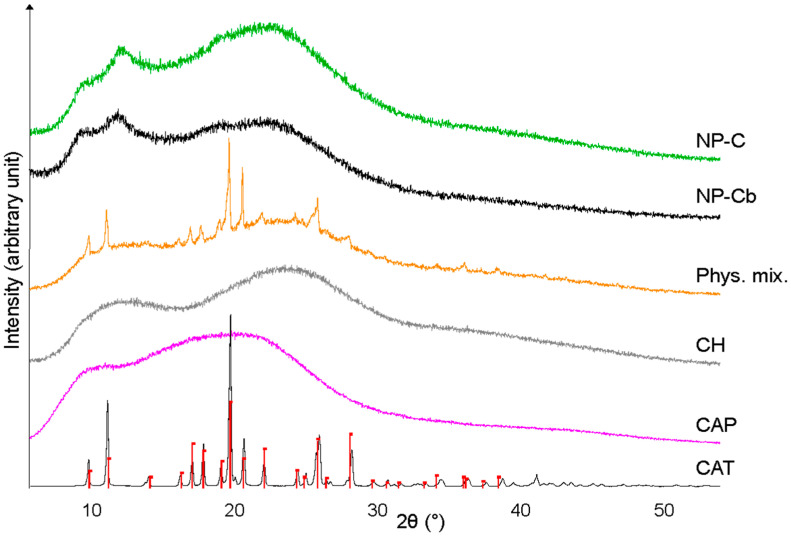
XRD patterns of CAT, CAP, CH, physical mixture, NP-Cb and NP-C; red bars: Powder Diffraction File 35–1916 (captopril).

The peaks in the pattern of CAT coincide with Powder Diffraction File No. 35–1916 of captopril (Figure 5), which crystallises in the orthorhombic system, space group P2_(1)_2_(1)_2_(1)_ [38]. CAP is an amorphous material, in agreement with literature data [39,40]. In fact, only humps (at 11.0, 19.0, 20.5 and 43.0°) are detectable in the diffractogram, which is also the case of CH (Figure 5), whose X-ray pattern (with humps at 12.0, 24.0 and 36.0°) traces that reported by Rassu et al. for chitosan [41]. The diffractogram of the physical mixture (Figure 5) combines the peaks of CAT with a background that reproduces the pattern of CH, while the CAP component is not as evident. NP-Cb and NP-C are amorphous and show similar patterns (Figure 5), with humps located at 9.7, 12.0, 19.0 and 22.5°. The absence of captopril peaks in NP-C demonstrates that the drug contained in the NPs does not have a crystalline structure; this means that the preparation process of the nanoparticles has determined captopril amorphisation. On the other hand, the production method and the interaction with other components can vary the structure of captopril, as recently reported by Musuc at al. for a captopril–cyclodextrin complex [42]. Although the amorphous form is more soluble than the crystalline form [43], an increase in CAT solubility was not expected as CAT is a water-soluble drug. Furthermore, not even an increase in the dissolution rate of amorphous CAT was obtained, as confirmed by the results obtained from the in vitro drug release study.

No change in the therapeutic properties of CAT is expected after the administration of NP-C. de Azevedo and co-workers administered in vivo amorphous CAT, complexed by cyclodextrin: the reduction in mean arterial pressure was similar to that upon crystalline CAT administration [44].

#### 2.5.3. Thermal Analysis (TG-DTG-DTA)

**Figure 6:**
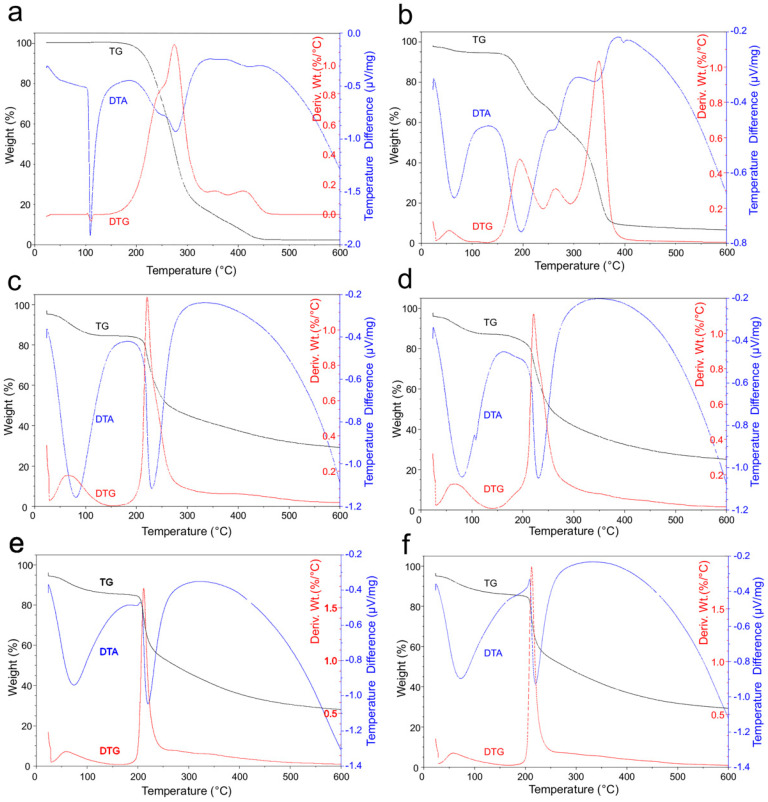
TG, DTG and DTA curves of: (**a**) CAT; (**b**) CAP; (**c**) CH; (**d**) Physical mixture; (**e**) NP-Cb; (**f**) NP-C.

The DTA curve of CAT shows a sharp endothermic peak at 109 °C, determined by the melting of captopril (Figure 6a), followed by four endothermic reactions (at about 242, 277, 360 and 422 °C), which correspond to weight losses, clearly visible along the DTG curve, related to the progressive decomposition of the drug. The residual mass at the end of the analysis is 2.5% (see TG curve in Figure 6a). All data match those reported by Musuc et al. and Huang et al. [42,45].

The DTA of CAP in Figure 6b shows four peaks at approximately 60, 194, 260 and 348 °C due to the weight lost at the same temperatures (see DTG peaks), followed by a last slight endotherm at 395 °C that should be related to a phase transition occurring in the residual mass (about 8%). Similar results were obtained by Roxin et al. for cellulose acetate phthalate [40]. 

The DTA curve of CH shows two strong endothermic reactions at 80 and 230 °C (Figure 6c). The first is due to the loss of water physically adsorbed and/or weakly hydrogen-bonded to the polymer (peak at ≈70 °C on the DTG), and the second (at 221 °C) to chitosan decomposition [46], which left 29% of residual mass at 600 °C.

Overall, the physical mixture displays curves similar to CH (Figure 6d). The presence of crystalline captopril is revealed by the weak but sharp melting endotherm at 107 °C along the DTA, while the faint peak at about 185 °C should correspond to the main endothermic reaction (related to a weight loss) of CAP.

The TG and DTG curves of NP-Cb and NP-C are superimposable (Figure 6e,f). Their profiles are similar to those of the physical mixture, although the main DTG peak appears sharper and exhibits a slightly lower temperature (212 °C vs. 221 °C). The DTA profiles of NP-Cb and NP-C are also similar (Figure 6e,f), except for the weak endothermic peak at 195 °C (due to CAP, see above), which is only noticeable in NP-Cb. NP-C contains the same amount of captopril as the physical mixture, but the drug in the nanoparticles is amorphous, this explains the absence of the captopril melting peak along the DTA of NP-C.

### 2.6. In Vitro Drug Release Study

**Figure 7:**
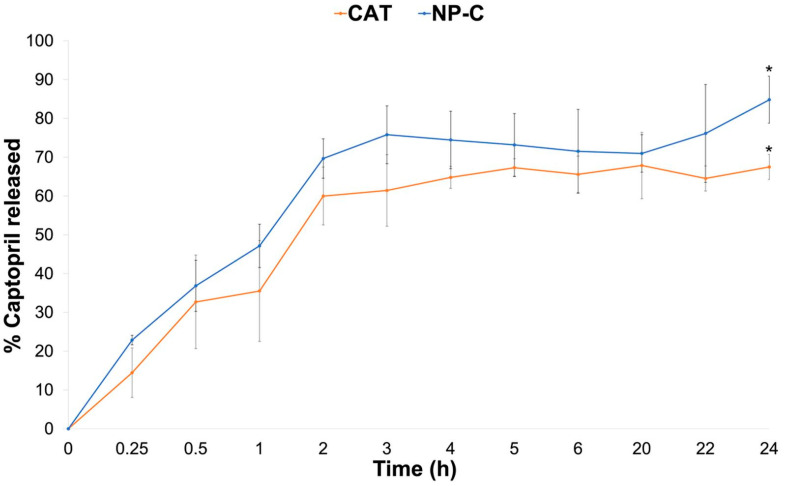
Comparison of the in vitro drug release profiles from the NP-C and CAT solutions. * *p* value < 0.05: CAT vs. NP-C after 24 h.

The in vitro drug release of NP-C dispersion (Figure 7) was performed using pH 7.4 phosphate buffer as medium to simulate the biological fluid regardless of the administration route. CAT is released from NP-C with linear kinetic (R^2^ = 0.983) from 0 to 2 h, reaching 70% of the amount tested after 2 h and 84.81 ± 6.07% after 24 h. CAT from the CAT solution, in comparison, reached 67.47 ± 3.24% of the amount expected at the end of the test; statistical analysis of 24 h release data indicated a significant difference (*p* < 0.05). Lag time for release from the dispersion was not observed due to the amount of free-CAT in the dispersion. These results demonstrated that NP-C in contact with pH 7.4 gradually dissociates due to the changes in the charges of both polymers and CAT resulting in CAT release [30]. This behaviour was also confirmed by the size analysis of NP-C at different pHs: as reported in Figure S2, the mean diameter decreased as the pH of the medium increased from 3 to 7, confirming the pH dependent of the interpolymer complex. In fact, NP preparation takes place at pH 3, where the amino groups of CH are protonated, so the CH chains from the surface of the nanoparticles are in an extended conformation. At pH = 7.4, the carboxylic groups of CAP are dissociated, but the CH is in neutral form, so the interpolymeric complex dissociates. This dissociation leads to the disappearance of the NPs, and any possible toxicity of the nanodelivery system for children is prevented.

### 2.7. In Vitro Cytotoxicity

**Figure 8:**
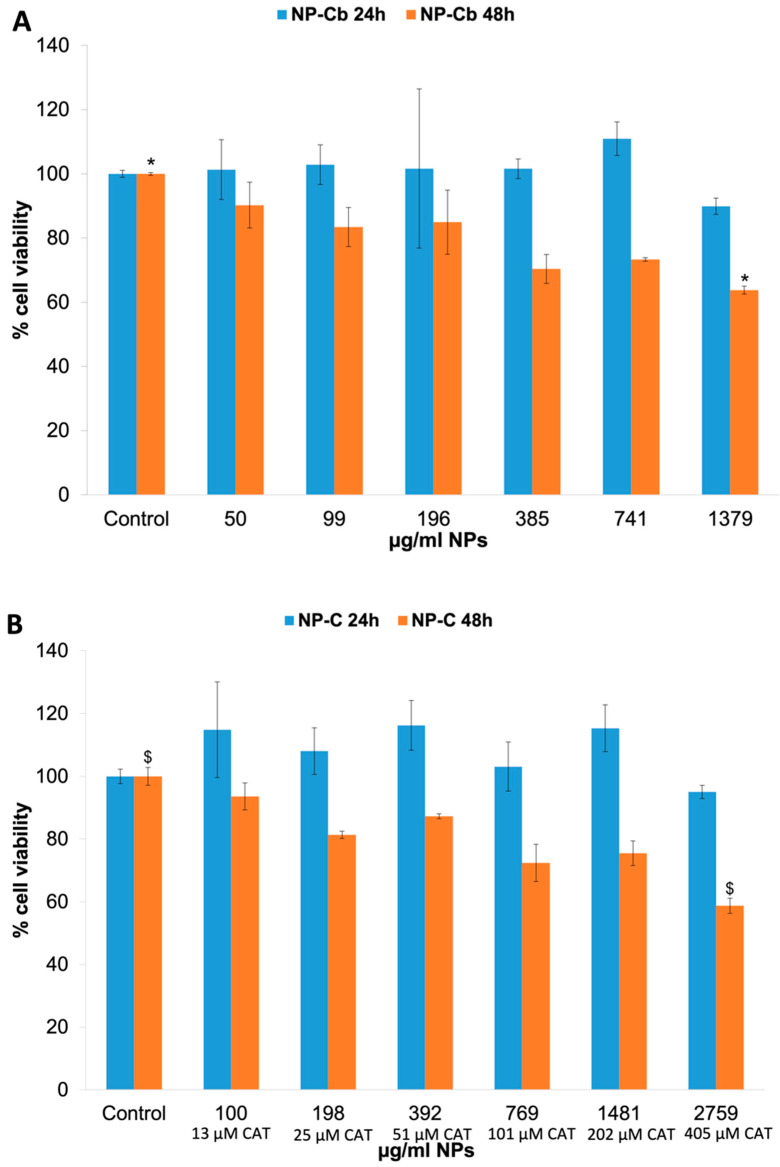
In vitro cytotoxicity. (**A**) Effect of NP-Cb on HFF-1 cells after 24 h (blue bars) and 48 h (orange bars) of incubation: * *p* value < 0.05: the effect of 1.379 μg/mL NP-Cb vs. control. (**B**) Effect of NP-C on HFF-1 cells after 24 (blue bars) and 48 h (orange bars) of incubation: $ *p* value < 0.05: the effect of 2.759 μg/mL NP-C vs. control.

Cytotoxicity studies of the NP-C dispersion were performed on neonatal human fibroblasts as this cell line is considered an important target for NP toxicity studies [47]. As shown in Figure 8, HFF-1 cells treated for 24 h with NP-Cb (50–1380 μg/mL) or NP-C (100–2759 μg/mL) equivalent to 13, 25, 51, 101, 202, 405 μM CAT concentrations did not show significant cytotoxic effects compared to the control groups of DMSO alone (*p* > 0.05). In fact, the viability >70% is considered as “no toxicity”. After 48 h, NP-Cb at the highest concentration (1380 μg/mL) and NP-C (2759 μg/mL) equivalent to 405 μM CAT slightly reduced cell viability by around 64% and 59%, respectively, indicating dose-dependent cytotoxicity (*p* < 0.05). No significant differences in cytotoxicity were found between NP-Cb and NP-C after 24 and 48 h (*p* > 0.05). Free-CAT (equivalent to 100 μM and 1000 μM) did not display changes on HFF-1 cells viability after 24 h, whereas after 48 h, cell viability was reduced to 61% for a CAT concentration of 100 μM and 65% for 1000 μM (Figure S3). Even so, there was no statistically significant difference after 48 h (*p* > 0.05).

As reported by other authors, CH, rather than being a cytotoxic compound, is a polymer that contributes to increasing cell viability [48]. A previous study displayed that the toxicity of NPs in fibroblast cells was low [49]. Additionally, Ray et al. showed that fibroblast viability decreased minimally in a stepwise manner as the nanoparticle concentration increased [50]. Furthermore, according to a review of this natural polysaccharide and current studies, it could be surmised that, in the appropriate conditions, chitosan exhibits low toxicity in the μg/mL range. At therapeutic doses, chitosan’s in vivo toxicity was shown to be largely benign. Although some toxicity was found at high doses, the researchers determined that chitosan was safe for in vivo use [51].

Assuming drug therapeutic concentrations from clinical studies might approach the 1 mg/L range (5 μM), these concentrations match NP concentrations in the low range of Figure 8, hence NP-C would not be very toxic in vivo. It is necessary to consider that an in vitro system approach is used as a preliminary screen for NP-C, but more studies are needed to characterise and compare the in vivo toxicity of the formulation for clinical use.

## 3. Materials and Methods

### 3.1. Materials

Captopril (CAT, ε98% HPLC) and cellulose acetate phthalate (CAP, Mw: 2534.12 g/mol) were purchased from Sigma-Aldrich (St. Louis, MO, USA). Chitosan chloride (CH, Protasan UP CL 113, Mw: 160,000 g/mol, deacetylation degree, 86%) was obtained from Novamatrix (Drammen, Norway). Acetone was bought from Carlo Erba Reagents (Val de Reuil, France). Ultrapure bi-distilled water was obtained by a MilliQ R4 system, Millipore (Milan, Italy). Acetonitrile and ethanol of chromatographic grade were purchased from Merck (Darmstadt, Germany). Hydrochloric acid 37% was acquired from Honeywell Fluka^TM^ (Seelze, Germany). Dulbecco’s modified Eagle’s medium (DMEM), 10% fetal bovine serum (FBS) and 3-(4,5-dimethyl-thiazol-2-yl)-2,5,diphenyltetrazoliumbromide (MTT) were acquired from Merck (Madrid, Spain). HFF-1 cells were obtained from the ATCC (Barcelona, Spain).

### 3.2. Preparation of Polymeric Nanoparticles

**Figure 9:**
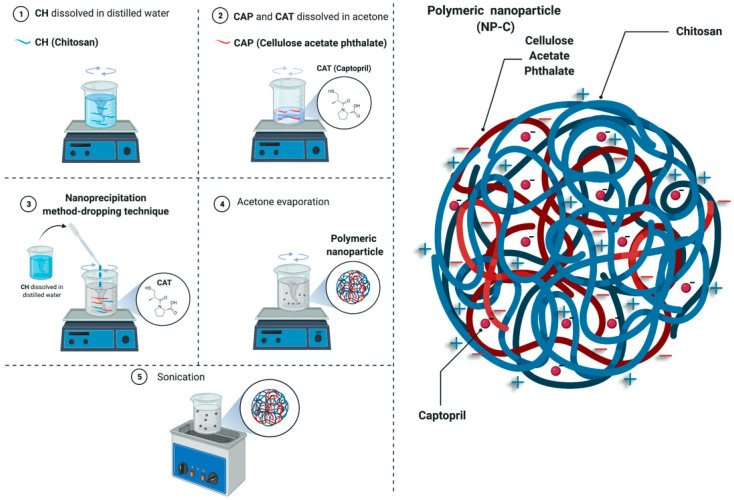
Nanoprecipitation method–dropping technique. Created with BioRender.com.

Drug-free and loaded nanoparticles were prepared, without using surfactants, by the nanoprecipitation method/dropping technique (Figure 9) [52]. The polymer used was either CAP alone (NP-Ab and NP-A, respectively) or CAP with CH at a 1:1 *w*/*w* ratio (NP-Bb and NP-B) or 1:3 *w*/*w* (NP-Cb and NP-C) ratio (Table 1). NPs consisting of CH and CAT alone cannot be obtained with this technique. CAP (50 mg) was dissolved in 5 mL of acetone under magnetic stirring to provide the organic phase. The aqueous phase was 20mL Milli Q water pH 3, acidified with hydrochloric acid 0.01% (calibrated pH meter, Eutech Instruments, pH 510, Singapore). Where CH was used, it was solubilised in the aqueous phase. Ten milligrams of CAT were dissolved in the acetone along with the CAP to prepare loaded NPs (NP-A, NP-B, NP-C). Then, the aqueous phase was added drop by drop to the organic phase under mild magnetic stirring. Acetone was evaporated for 2 h in a water bath at 60 °C under magnetic agitation. After that, the rotary evaporation method (Rotavapor RE111, Bünchi Labortechnik AG, Flawil, Switzerland) at 60 °C under vacuum for 5 min was used to remove any remaining acetone. Finally, the dispersion was sonicated in an ultrasonic bath for 40 s (60/120 W and 35 kHz, Bandelin sonorex RK52H, Heinrichstrabe, Berlin, Germany).

This preparation method was defined based on the pre-formulation studies reported in the Supplementary Materials.

NPs prepared were directly characterised both in dispersion and after isolation by ultrafiltration for solid state characterisation as described in Section 3.9.

### 3.3. Particle Size, Polydispersity Index and Zeta Potential Measurements

Mean particle size and PDI were measured by the dynamic light scattering method using a Coulter Submicron Particle Sizer N5 (Beckman-Coulter Inc. Miami, Florida, USA). The measurements were performed after dilution of NP dispersion in MilliQ water adjusted to pH 3 (with 0.01% hydrochloric acid) previously filtered (regenerated cellulose syringe filter, pore size: 0.20 μm, filter size: 15 mm, Albet LabScience, Dassel, Germany). Triplicate NP formulations were prepared, and each sample was analysed three times (*n* = 9). 

Zeta potential of NP dispersions was evaluated in Malvern Zetasizer Nano ZS ZEN 3600 (Malvern Instrument, Worcestershire, UK).

The particle size of NP-C dispersion was also measured using dispersion media at different pHs. The measurements were performed immediately after dilution of NP-C dispersions in Milli Q water acidified to pH 5 with 0.01% hydrochloric acid and in water pH 7 previously filtered as reported above.

### 3.4. Quantitative Analysis of Captopril

For quantitative determination of CAT, a rapid and sensitive high-performance liquid chromatographic (HPLC)-modified method was used [53]. A Varian HPLC–DAD system (Palo Alto, CA, USA) consisting of two ProStar 210 pumps, a ProStar 410 autosampler and a DAD Varian 330 detector was employed. Varian workstation version 6.2 software was used for data acquisition and processing. The chromatographic separation was performed on a Water Spherisorb Nucleosil C_8_ column (250 mm × 4.6 mm ID and 5 μm of particle size, Supelco, Milan, Italy). The binary mobile phase consisted of acetonitrile and water pH 2.0 (45:55 *v*/*v*) (per H_3_PO_4_) was filtered through 0.22 μm cellulose regenerated membrane filters (Sartorius, Goettingen, Germany) prior to use and was pumped at a flow rate of 1.5 mL/min at room temperature. The injection volume was 10 μL and the analysis time was 3 min per sample. Peak areas in the chromatography were recorded and measured at 205 nm. Concentrations of CAT were correlated through a calibration curve (y = 70,963,714x − 41,338; R^2^ = 0.9999) obtained from standard solutions of drug (range 0.005–0.2 mg/mL) in phosphate-buffered solution 7.4.

### 3.5. Total Drug in Dispersion and Encapsulation Efficiency

The total drug in dispersion was determined. A dispersion volume (0.5 mL) was diluted in 2 mL of acetonitrile in a 5 mL volumetric flask and sonicated in an ultrasonic bath for 10 min. After that, 3 mL of 0.1% (*v*/*v*) phosphoric acid aqueous solution was added to assure an acidic pH and avoid the formation of disulphide bridges; the dispersion was sonicated for 10 min to extract the drug. The sample was centrifugated at 14,000 rpm, and the supernatant was analysed by HPLC as described in the supplementary data. Then, the total drug in dispersion was calculated as a percentage with respect to the amount of CAT used for preparing NPs.

Furthermore, the assessment of encapsulation efficiency was performed by quantification of the free drug separated from NPs by ultrafiltration with Amicon Ultra-15 centrifugal filters at 4400 rpm (Eppendorf Centrifuge 5702 R, Hamburg, Germany) for 10 min at 4 °C. The non-encapsulated drug in the filtrate was measured by HPLC and the intensity check using the Particle Sizer N5 was also evaluated. To validate the centrifugal ultrafiltration method, a CAT solution was centrifuged, and the filtrate was collected for HPLC assay. As a result, it was confirmed that absorption/adsorption of the drug to the membrane filter or any NPs in the filtrate did not occur. Encapsulation efficiency (EE) was calculated according to Equation (1):

(1) $$\mathrm{EE}\left(\%\right)=\frac{\mathrm{total}\;\mathrm{CAT}\;\mathrm{in}\;\mathrm{dispersion}\;-\;\mathrm{free}\;\mathrm{CAT}}{\mathrm{total}\;\mathrm{CAT}\;\mathrm{in}\;\mathrm{dispersion}}\times 100$$

The results obtained are the average of three NP formulations analysed 2 times (*n* = 6).

### 3.6. Morphological Analysis

The morphology of NP-Cb and NP-C dispersion was observed with JSM-IT500 InTouchScope™ Scanning Electron Microscope (SEM) (Jeol Ltd., Akishima, Tokyo). SEM studies were carried out on sputter gold-coated NP pellets (Polaron E-5000) obtained after dilution, centrifugation and gradual drying on top of aluminium stubs.

### 3.7. Physical Stability

The physical stability of NP-Cb and NP-C dispersion was studied by analysing the mean diameter and PDI, as reported in the Section 2.3, at 1, 7, 14 and 28 days after preparation under different storage conditions (4 °C and 25 °C). The test was carried out on three NP formulation samples. Each sample was analysed twice (*n* = 6).

### 3.8. Chemical Drug Stability

The chemical stability of CAT in NP-C dispersion and aqueous solution at pH 3 was evaluated for 1, 7, 14 and 28 days under different storage conditions (4 °C and 25 °C). The total CAT was assessed by HPLC as reported in the Section 2.5. Values are the mean of three independent analyses (*n* = 6).

### 3.9. Solid State Characterisation

NP-Cb and NP-C were isolated from dispersion by ultrafiltration with Amicon Ultra-15 centrifugal filters at 4400 rpm (Eppendorf Centrifuge 5702 R, Hamburg, Germany) for 10 min at 4 °C and dried at room temperature before the analysis of the chemical and crystalline structures as well as thermal behaviour.

#### 3.9.1. Fourier Transform Infrared Spectroscopy (FTIR) Analysis

Sample discs (NP-Cb, NP-C, physical mixture and CAT) were prepared by intimately mixing about 1 mg of each one with 100 mg of potassium bromide using a pestle and mortar and compressing the mixture in a 15T Manual Hydraulic Press. Fourier transform infrared spectroscopy (FTIR) was performed on a Nicolet Avatar 320 FTIR spectrometer (Nicolet Instrument Corporation, Madison, WI, USA) equipped with EzOminic version 6.0 software (Ezonics, Fremont, CA, USA).

#### 3.9.2. X-ray Diffraction (XRD) Analysis

X-Ray diffraction (XRD) was performed using a diffractometer Bruker D2 Phaser (Billerica, MA, USA) equipped with a Cu tube and a LynxEye PSD detector (angular opening 5°). Working conditions were as follows: 30 kV, 10 mA, scanning in the 2θ range of 5.8–60°, step size of 0.02°, counting time of 1 s per step and spinner at 15 rpm. Bruker low background Si-crystal specimen holders were employed. Bruker EVA 14.2 software (Billerica, MA, USA) and the PDF-2 (ICDD) database were used to evaluate the X-ray patterns.

#### 3.9.3. Thermal Analysis (TG-DTG-DTA)

A TA Instrument Q600 (CeSAR—Centro Servizi di Ateneo per la Ricerca, Sassari University) was used to perform thermogravimetric (TG), derivative thermogravimetric (DTG) and differential thermal analysis (DTA). The samples, about 10 mg in weight, were brought from room temperature (approx. 17 °C, RH approx. 50%) to 24 °C with a gradient of 1 °C/min, held for 10 min at this temperature, and then heated to 600 °C with a rate of 10 °C/min. Analyses were carried out under a nitrogen flow of 100 mL/min and using Al_2_O_3_ crucibles (an empty crucible was used as a reference). The results were evaluated through the TA-Universal software.

### 3.10. In Vitro Drug Release Study

In vitro drug release test of NP-C dispersion was performed in pH 7.4 phosphate buffer (100 mL) using the dialysis bag method at 37 °C, under stirring at 100 rpm, assuring sink condition [54]. Cellulose acetate dialysis bags (12,500 cut-off, Sigma-Aldrich St. Louis, MO, USA) were filled with 2 mL of NP-C dispersion or CAT solution (0.5 mg/mL) as a comparison. At predetermined time intervals (0.25–24 h), samples of acceptor fluid were withdrawn and replaced by equal volumes of fresh buffer. The quantification of CAT released was assayed by HPLC. The release studies were performed in triplicate.

### 3.11. In Vitro Cytotoxicity

Neonatal human dermal foreskin fibroblast-1 (HFF-1) cells were procured from ATCC (American Type Culture Collection) and grown in Dulbecco’s modified Eagle’s medium (DMEM) and fetal bovine serum (FBS) to a final concentration of 15%. The cells were maintained at 37 °C in a humidified 5% CO_2_ incubator using standard cell culture procedures. The cytotoxicity effect of NPs on the HFF-1 cell line was determined by MTT assay. Cells were seeded at 15,000 viable cells/well density in 24-well plates with the medium containing 15% FBS and incubated for 48 h at 37 °C and 5% CO_2_. Later, cells were incubated (*n* ≥ 4) for 48 h with different concentrations of free-CAT aqueous solution (equivalent to 100 μM and 1000 μM) and variable volumes (2.5–80 μL) of NP-Cb and NP-C dispersion equivalent to 13, 25, 51, 101, 202, 405 μM CAT concentrations and 50–1380 μg/mL and 100–2759 μg/mL for NP-Cb and NP-C dispersion, respectively. After the treatment and incubation period, 10 μL of MTT was added to each well and incubated for 4 h. Purple colour formazan crystals formed were then dissolved in DMSO. Solution absorbance was determined at 570 nm, with a 690 nm reference wavelength, employing an ELISA reader. Cells treated with 5 μL DMSO were used as a control. Cell viability was calculated using the following Equation (2):

(2) $$\%\;Viability=\frac{Abs\;sample\times 100}{Abs\;control}$$

### 3.12. Statistical Analysis

Statistical analyses were exported to the GraphPad Prism 8.0 software (GraphPad Software, Inc., San Diego, CA, USA). Unpaired t-test was performed for the statistical difference between two treatment groups and one-way ANOVA analysis of variance test followed by a Tukey’s multiple comparison test in case of multiple comparisons. Statistical significance was set at *p* < 0.05.

## 4. Conclusions

The present study demonstrated that nanoparticles based on CAP and CH can be prepared by exploiting only the interaction between the two polymers without using surfactants. The loading of the hydrophilic drug CAT was affected by the polymer weight ratio, and a high amount of CH was necessary for obtaining a good encapsulation efficiency. NP-C had a spherical shape and good physical stability in dispersion thanks to the high zeta potential. Furthermore, NP-C protected CAT, both encapsulated and free, from chemical degradation in water. When in contact with fluids with physiological pH, the polymer matrix gradually dissociated and released CAT. The nanoparticles prepared did not show in vitro cytotoxic effects except at the highest dose tested after 48 h. Therefore, NP-C can be proposed as a platform for preparing different dosage forms for CAT delivery in paediatrics. Nanoparticle dispersion could be used for preparing (i) oral formulation by adding, e.g., sweeteners and flavourings to improve palatability, (ii) enteral formulation and (iii) intravenous or parenteral injection. For applicability, further studies are necessary.

## Figures and Tables

**Table 1:** Composition and physicochemical properties of the NPs.

Formulation	Drug	Polymer	Size (nm)	PDI	Total Drug in Dispersion (%)	Encapsulation Efficiency (%)
NP-Ab	-	CAP	259.2 ± 30.0 ^Δ^	0.10 ± 0.07 ^•^	-	-
NP-Bb	-	CAP:CH 1:1 *w*/*w*	252.5 ± 16.4 ^Δ^	0.21 ± 0.09 ^•^	-	-
NP-Cb	-	CAP:CH 1:3 *w*/*w*	478.6 ± 21.1 *^,Δ^	0.16 ± 0.06	-	-
NP-A	CAT	CAP	259.8 ± 27.7 ^¥^	0.06 ± 0.03 ^§^	103.9 ± 3.2	11.7 ± 8.8 ^#^
NP-B	CAT	CAP:CH 1:1 *w*/*w*	248.3 ± 31.9 ^¥^	0.23 ± 0.04 ^§^	100.3 ± 8.0	32.6 ± 2.3 ^#,$^
NP-C	CAT	CAP:CH 1:3 *w*/*w*	427.1 ± 32.7 *^,¥^	0.17 ± 0.09 ^§^	99.16 ± 0.5	61.0 ± 6.5 ^#,$^

* *p* value < 0.0001: NP-Cb vs. NP-C. ^¥^ *p* value < 0.0001: NP-C vs. NP-A and NP-B. ^Δ^ *p* value < 0.0001: NP-Cb vs. NP-Ab and NP-Bb. ^§^ *p* value < 0.05: NP-A vs. NP-B and NP-C. ^•^ *p* value < 0.05: NP-Ab vs. NP-Bb. # *p* value < 0.001: NP-A vs. NP-B and NP-C; ^$^ *p* value < 0.0001: NP-B vs. NP-C.

## Data Availability

Data are contained within the article and supplementary material.

## Supplementary Materials

The following supporting information can be downloaded at: https://www.mdpi.com/article/10.3390/ph15060662/s1; Table S1: Influence of the solvent of CAT in CAP NPs. Figure S1: Influence of storage (4 °C and 25 °C) on mean diameter of NP-Cb (A) and NP-C (B) over 28 days (*n* = 6). Figure S2: Influence of pH of dispersion media on particle size (A) and PDI (B) of NP-Cb and NP-C. Figure S3: In vitro cytotoxicity. Effect of free-CAT (100 μM and 1000 μM) on HFF-1 cells after and 48 h of incubation.

## Artwork

Graphical abstract and Figure 9 were created with BioRender.com.
