# Supplementary material for: Surfactant-Free Chitosan/Cellulose Acetate Phthalate Nanoparticles: An Attempt to Solve the Needs of Captopril Administration in Paediatrics
Source: Pharmaceuticals (Basel). 2022 May 25;15(6):662. doi: 10.3390/ph15060662 (PMC9228859; doi:10.3390/ph15060662)
Supplement: Supplementary file 1 [file pharmaceuticals-15-00662-s001.zip › pharmaceuticals-1716712-supplementary.pdf]

Supplementary Materials

Surfactant-free nanoparticles made of chitosan-cellulose acetate phthalate interpolymer complex as carrier of captopril in Paediatrics

Noelia Nieto González [1], Guido Cerri [2], Jesús Molpeceres [3], Massimo Cossu [4], Giovanna Rassu [5,*], Paolo Giunchedi [5], Elisabetta Gavini [5]

[1] PhD program in Chemical Science and Technology, Department of Chemistry and Pharmacy, University of Sassari, 07100 Sassari, Italy. nnietogonzale@uniss.it

[2] Department of Architecture, Design and Urban Planning - GeoMaterials Lab, University of Sassari, 07100 Sassari, Italy. gcerri@uniss.it

[3] Department of Biomedical Sciences, Faculty of Pharmacy, University of Alcalá, 28805 Alcalá de Henares, Spain. jesus.molpeceres@uah.es

[4] Department of Chemistry and Pharmacy, University of Sassari, 07100 Sassari, Italy. mcossu@uniss.it

[5] Department of Chemistry and Pharmacy, University of Sassari, 07100 Sassari, Italy. Present address: Department of Medical, Surgical and Experimental Sciences, University of Sassari via Muroni 23a - 07100 Sassari. grassu@uniss.it; pgiunc@uniss.it; eligav@uniss.it

* Correspondence: grassu@uniss.it; Tel.: +39 079 228735

1. *Methods*

1.1. *Pre-formulation studies*

The amount of CAP to use for the preparation of NPs were studied. Firstly, 20 mg (CAP 20) or 50 mg (CAP 50A) of CAP were dissolved in 5 ml of acetone under magnetic stirring and then 15 ml of Milli Q water was added drop by drop (Hornig & Heinze, 2008). Acetone was evaporated for 2 h at 60 °C under magnetic stirring. After that, the rotary evaporation (Rotavapor RE111, Bünchi Labortechnik AG, Flawil, Switzerland) at 60°C for 5 min was used to remove any remaining acetone. Finally, the dispersion was sonicated in an ultrasonic bath for 40 s and analysed by dynamic light scattering method (Coulter Submicron Particle Sizer N5, Beckman-Coulter Inc. Miami, Florida, USA). Also, CAP 50B was prepared under the same conditions as CAP 50A, but 20 ml of Milli Q water was added drop by drop in the organic phase.

Furthermore, the optimal solvent of CAT was evaluated. CAT was dissolved in water (CAP 50B1) or in acetone (CAP 50B2); then, the preparation method was the same.

The evaporation method to eliminate the organic solvent was also assessed: evaporation was performed at room temperature for 4 h or for 2 h in a water bath at 60 °C both under magnetic stirring (200 rpm).

2. *Results*

2.1. *Pre-formulation studies*

CAP 20 and CAP 50A showed a mean particle size of 279.1±3.3 nm and 457.4±6.0nm and a PDI of 0.01±0.09 and 0.14±0.09, respectively. However, CAP 20A dispersion displayed a slight opalescence and a low NP concentration: therefore, the high CAP amount was selected.

CAP 50B, prepared with 20 ml of Milli Q water exhibited a lower mean size, 248.4±1.2 nm and PDI of 0.070±0.003 than CAP 50A.

Results of CAT dissolved in water or acetone (CAP 50B1 and CAP 50B2) are shown in Table 1S. Formulation studies showed that CAP 50B1 have no loading capacity compared to CAP 50B2.

CH1 evaporated at room temperature for 4 h showed a mean size of 449.1±47.5 nm, a PDI of 0.12±0.01 and encapsulation efficiency of 51.6±3.0 %.

Furthermore, the best evaporation method resulted the evaporation for 2 h in a water bath at 60 °C allowed to obtain reproducible results.

Table S1. Influence of the solvent of CAT in CAP NPs.

Formulation	Drug	Polymer	Size (nm)	PDI	Encapsulation Efficiency (%)
**CAP 50B1**	CAT in water	CAP	263.1±2.9	0.076±0.017	0
**CAP 50B2**	CAT in acetone	CAP	245.0±3.1	0.100±0.003	10.2±1.5

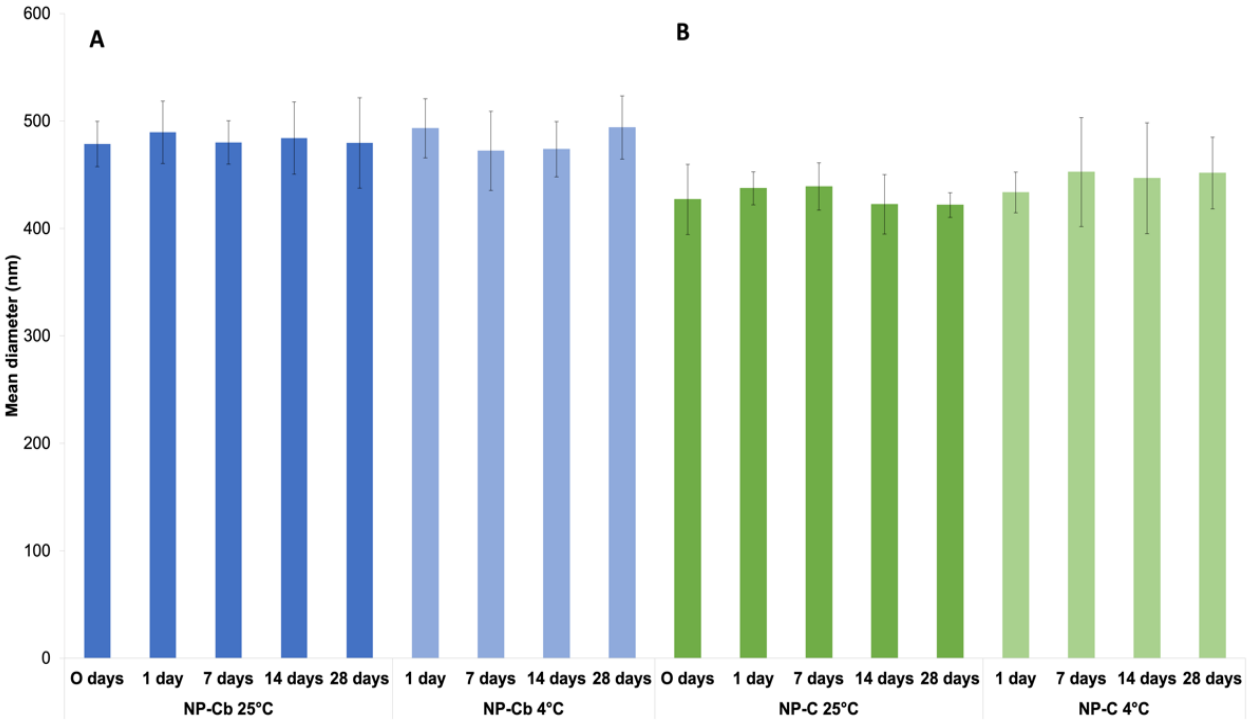

Figure S1. Influence of storage (4° and 25°C) on mean diameter of NP-Cb (A) and NP-C (B) over 28 days (n = 6).

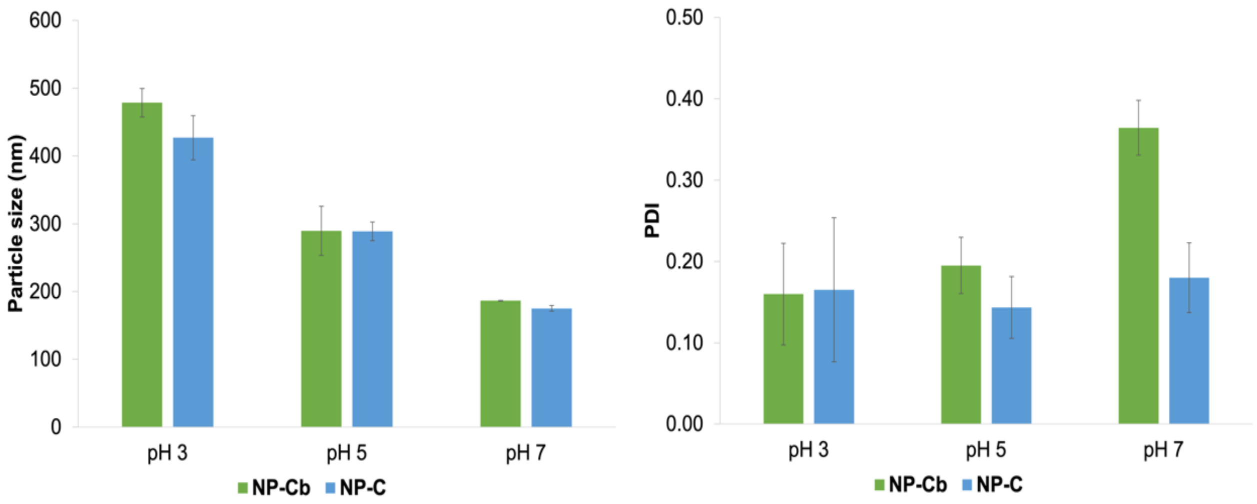

Figure S2. Influence of pH of dispersion media on particle size (A) and PDI (B) of NP-Cb and NP-C.

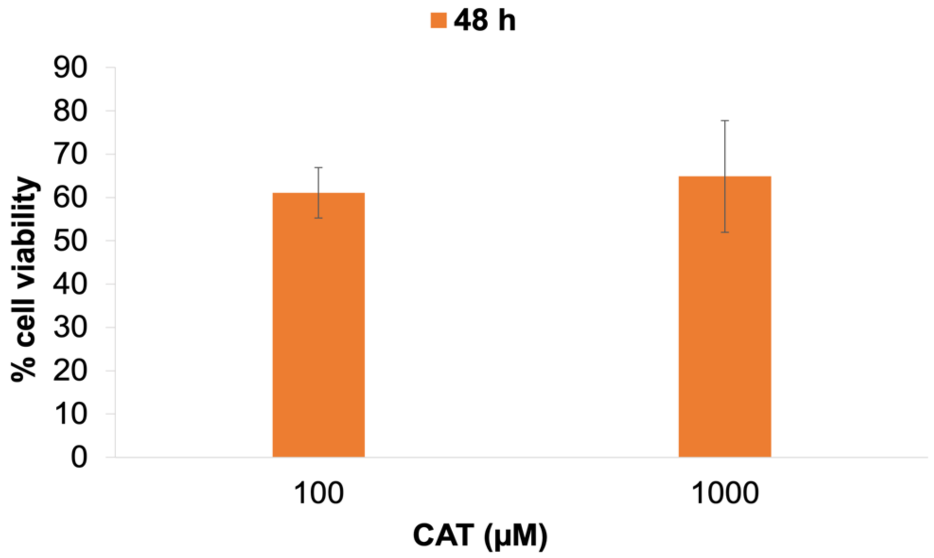

Figure S3. *In vitro* cytotoxicity. Effect of free-CAT (100 μM and 1000 μM) on HFF-1 cells after and 48 h of incubation.
